# Advancing microalgae biomass cultivation for an integrated sustainable wastewater treatment and resource recovery

**DOI:** 10.1016/j.isci.2026.116435

**Published:** 2026-06-29

**Authors:** Akansha Shrivastava, Vishal Mishra, Divya Rishi Shrivastava

**Affiliations:** 1 Department of Biosciences, Manipal University Jaipur, Jaipur, Rajasthan 303007, India; 2 School of Biochemical Engineering, Indian Institute of Technology (Banaras Hindu University), Varanasi 221005, India; 3 Department of Electrical Engineering, Manipal University Jaipur, Jaipur, Rajasthan 303007, India

**Keywords:** applied sciences, environmental science, environmental technology

## Abstract

Microalgae have emerged as a promising bioresource for the integrated treatment of effluent, the production of biomass, and the mitigation of carbon emissions, thereby contributing to the Sustainable Development Goals (SDGs 7 and 13). This review summarizes the most recent developments in the management of wastewater using microalgae, with a particular focus on resource recovery and process integration. Nevertheless, challenges such as oxygen accumulation, biofouling, high energy demand, and scale-up constraints persist, despite the fact that configurations such as tubular, flat-panel, and bubble-column reactors offer flexibility across scales. Additionally, the topic of the integration of artificial intelligence and machine learning is addressed in order to address process constraints by means of predictive modeling, real-time optimization, and enhanced strain and process selection. In conclusion, this review offers a concentrated viewpoint on the advancement of sustainable microalgae systems by integrating technological and biological innovations.

## Graphical abstract

**Figure:**
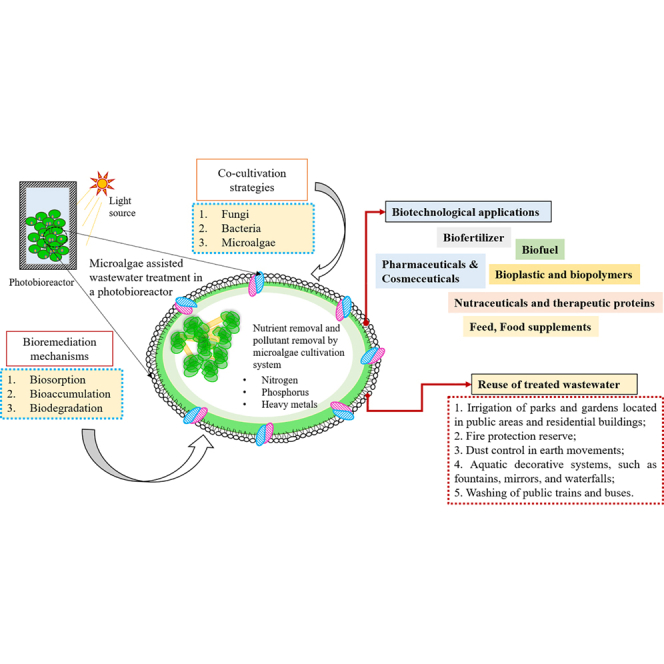

## Introduction

Water is an important source and feedstock in a variety of spheres, including agrochemicals, drinks, electronics, food, petrochemicals, pharmaceuticals, the oil and gas sector, and domestic usage.^1^ Because of the multiple chemicals it contains and the serious environmental implications it raises, there is growing concern over the direct dumping of contaminated water from these applications. Certain substances present in effluent are highly detrimental to living organisms. The chemical oxygen demand (COD) and biological oxygen demand (BOD) indicates the discharge of substantial quantities of organic and inorganic nutrients into the environment. Solid waste formation and undesirable emissions into the atmosphere are among the environmental challenges that eutrophication of aquatic ecosystems, which is exacerbated by excessive phosphorus and nitrogen loadings, induces.^2^^,^^3^ Additionally, it exacerbates pre-existing health concerns in the vicinity of the discharge site by facilitating the dissemination of adverse microbes that harm other aquatic species and degrade the quality of drinkable water.^4^ The most frequently identified contaminants in wastewater are heavy metals (HMs).^5^ Direct inhalation, ingestion, and contact with these hazardous compounds can result in severe health issues and elevate the likelihood of developing cancer, even at extremely low concentrations.^6^

Municipal, industrial, agricultural, and storm water are the four categories into which wastewaters are classified.^7^^,^^8^ Agricultural effluent, which encompasses drainage water, swine, and poultry, is discharged by livestock ranches and agricultural lands. Pesticides, herbicides, manure, pathogens, antibiotics, and large levels of phosphate, nitrogen, and organic carbon are present.^9^ Municipal wastewaters contain microplastics from domestic sources, as well as contaminants of increasing apprehension such as personal care items and prescription medications, in addition to phosphate and nitrogen compounds. Various sources, including mining, food processing, power plants, textiles, and energy-related industries, can generate industrial effluent, which may contain HMs, phenol and dyes.^10^ In recent years, the frequency of the individuals who are concerned about the “emerging pollutants” in waterbodies, as well as the potential hazards and consequences they may have on aquatic life and human health, have increased. As a consequence of the development of more effective analytical techniques (i.e., lower detection limits) and the growing comprehension of their hazards, synthetic organic compounds have only recently been recognized as emerging contaminants, despite their extensive environmental presence.^11^^,^^12^ Artificial stimulants, insecticides, pharmaceutical compounds, flame retardants, and per and polyfluoroalkyl substances (PFASs) are among the numerous categories into which these new chemicals are classified. Currently, there is a dearth of exhaustive understanding vis-à-vis the adverse effects on the environment and the human health.^13^^,^^14^

In order to succeed the intended purification objectives, it is essential to select the appropriate treatment strategy when purification is necessary.^15^^,^^16^ A variety of physical and chemical treatments are employed in conventional techniques.^17^^,^^18^ Conversely, biological treatments depend on the metabolic processes of microorganisms that degrade and transform effluent pollutants into related gases and biomass. Consequently, the effluent’s COD and BOD and levels decrease, while its integrity is enhanced. For biological pretreatment, microorganisms such as fungi, bacteria, yeast, and microalgae, are implemented.^19^ Consequently, they are regarded as a more environmentally favorable solution than physicochemical alternatives. The primary objective of the extensive research on microalgae as potential bioremediation agents for tertiary wastewater treatment is to eliminate conventional pollutants, antibiotics, and pathogens.^20^^,^^21^^,^^22^ This is due to their remarkable metabolic adaptability. In addition to the absence of adverse consequences, the utilization of these bio agents offers an additional cost advantage that is highly appealing from an industrial perspective. Biochar, a substitute for coal-derived carbon in effluent treatment operations can be produced by microalgae biomass.^23^^,^^24^ Moreover, the tertiary treatment configuration design process is facilitated by the ability of the microalgae to grow in the bioreactors. Recently, microalgae-based methodologies for the managing wastewater from agro-industrial complexes, livestock, businesses, and cities have gained the interest.

Microalgae can lower eutrophication risk by removing phosphate and nitrogen components.^25^ These are considered to be adaptable biological treatment alternatives that transform detrimental inorganic and organic components into usable biomass. Furthermore, microalgae collected from specialized treatment ponds can be used for a variety of applications in a wide range of industries, including food and fuel. Numerous microalgae species have exhibited extraordinary capability for the bioremediation of nutrients, emerging contaminants (ECs), HMs, and pathogens from wastewater, including *Botryococcus*, *Chlorella*, *Scenedesmus*, *Phormidium*, *Limnospira* (previously *Arthrospira*, *Spirulina*), and *Chlamydomonas*.^26^^,^^27^ Several microalgae species flourished in wastewater, including *Ankistrodesmus*, *Chlorella*, *Chlamydomonas*, *Euglena*, *Scenedesmus*, and *Oscillatoria*. In addition to providing clean water, microalgae-based wastewater treatment may recover nutrients from municipal, industrial and agricultural wastewater.^28^^,^^29^^,^^30^ Several studies have demonstrated that microalgae strains are skillful of enduring the harsh conditions of municipal waste and contemporary industry. Complex environmental behavior is exhibited by ECs, which include pharmaceuticals, personal care products, microplastics, and antibiotic resistance genes (ARGs).^31^ This behavior is governed by physicochemical and biological processes, including adsorption, hydrolysis, photodegradation, redox reactions, and biodegradation. Adsorption onto suspended solids, sediments, and biomass particularly mediated by extracellular polymeric substances (EPSs) plays a critical role in the removal of hydrophobic and particulate ECs, including microplastics, through aggregation and sedimentation in aquatic systems.^32^ The degradation of light-sensitive compounds is further facilitated by oxidative transformation and photodegradation, while microbial metabolism facilitates the partial or complete biodegradation of specific organic contaminants. Nevertheless, conventional degradation pathways are unable to degrade a significant number of recalcitrant compounds, including carbamazepine, triclosan, and PFASs, resulting in their persistence and potential bioaccumulation.^33^ Furthermore, the toxicity of transformation products produced during treatment processes may be greater than that of their parent compounds, which raises additional environmental concerns.

The multifaceted function of biological treatment systems, particularly microalgae-based processes, in the removal of ECs has garnered attention.^34^^,^^35^ Through nutrient uptake, co-metabolism, oxygenation, and the production of reactive oxygen species, microalgae contribute to the degradation of contaminants and the suppression of microbial organisms that carry ARGs.^36^ Bioflocculation and entrapment within algal biomass can be used to remove microplastics, while ARG mitigation is achieved through changes in microbial community dynamics and reduced selective pressure (via antibiotic uptake/degradation). High-resolution mass spectrometry, GC-MS, and LC-MS/MS are among the advanced monitoring and analytical techniques that facilitate the detection of ECs.^37^ Their transformation products at ultra-trace levels, thereby facilitating a mechanistic understanding of their fate and transport. Real-time surveillance and predictive assessment are further enhanced by complementary tools, including biosensors, remote sensing, and artificial intelligence (AI)-driven models.^38^ Nevertheless, the accurate quantification and large-scale implementation of effective removal strategies are still subject to limitations due to the lack of standardized protocols, matrix complexity, non-target identification, and low contaminant concentrations.

**Figure 1:**
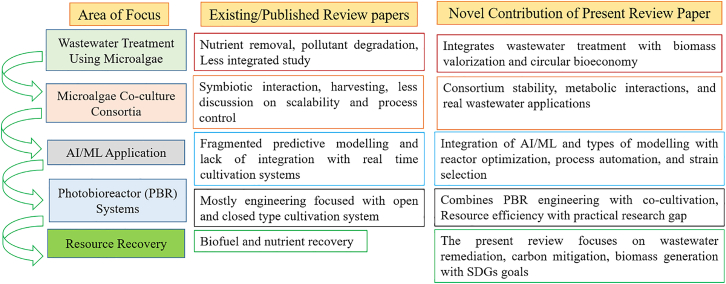
Comparative overview of existing literature and a novel contribution of the present review work^41^^,^^42^^,^^43^^,^^44^

Another alternative is to culture microalgae alongside other microbes to improve bioproducts output and wastewater treatment efficiency. In a co-cultivation approach, microalgae exchange nutrients and metabolites with heterotrophic microorganisms such as yeast, fungi and bacteria, increasing algal biomass production and improving bioremediation.^39^^,^^40^ The integration of AI and machine learning (ML) for process optimization introduces ethical concerns related to model transparency. The present review offers a comprehensive perspective that integrates advanced cultivation systems, microbial co-cultivation strategies, resource recovery pathways. Apart from this an emerging AI/ML-driven optimization approaches for sustainable microalgae-based wastewater treatment, in contrast to previous review articles that prioritize isolated aspects such as wastewater remediation efficiency, photobioreactor (PBR) engineering, or algal consortium development as mentioned in Figure 1.

And when coupled with internet of things (IoT)-based real-time monitoring systems it also deals with interpretability, algorithmic bias, data privacy, and accountability. In addition, the many types of growth systems such as closed, open, dark, and offshore their roles in the biomass production process are discussed. These critical variables could thus help researchers select the optimal biomass and alter the growing process and operational conditions to suit microalgae metabolism.

## Microalgae biomass in wastewater remediation

The extraction of primary nutrients from effluent, including phosphorus and nitrogen, as well as the treatment with variety of chemicals, has been successfully demonstrated by microalgae. In the context of HMs, it is imperative to recognize that microalgae are incapable of decomposing them rather, adsorb and store them within the cell ensemble.^45^ Due to their bioaccumulation, microalgae are considered a viable option for the development of biotechnologies for effluent treatment. When cultivated in effluent, microalgae have the capacity to ingest and sequester toxins, which can lead to a substantial decrease in pollutants. The growth and proliferation of microalgae result in the formation of larger clusters that are more easily removed and settled from treated water. The natural flocculation ability of microalgae enhances the efficacy and cost-effectiveness of biomass recovery, eliminating the need for artificial coagulants or other separation procedures.^46^ Furthermore, the effluent that has been entirely decontaminated is now suitable for a wide range of reuse applications, including irrigation, groundwater recharge and industrial operations. The microalgae’s assimilation of these compounds is contingent upon the species and its properties, which include the capacity to develop mixotrophically, chemical composition, rapid sedimentation, and endurance to severe temperatures.^47^ The absence of nutrients in the media may limit the proliferation of microalgae. Microalgae require carbon, nitrogen, phosphorus, and iron as essential nutrients for their proliferation.^48^

Nucleic acids, proteins, and cells are all composed of nitrogen, an essential chemical. The scarcity of total lipids in microalgae cells directly influences their synthesis and accumulation. During the cultivation phase, microalgae transform inorganic nitrogen to organic forms, thereby forming accessible in the medium. It is crucial to emphasize that specific cyanobacteria possess the distinctive capacity to fix nitrogen from the air, thereby replenishing their nitrogen source, whereas genuine microalgae rely on nitrogen from the medium.^49^ After purification, ammonium is the most prevalent nitrogen species in activated sludge effluent. According to research, microalgae prefer ammonium since it requires less energy to assimilate. Ammonia is the most frequently employed nitrogen source after ammonium is nearly depleted, followed by nitrates. In certain conditions, such as substantially high pH levels, ammonia (NH_3_) volatilization may occur in systems with temperatures above 30 °C, potentially resulting in decreased availability.^50^ Phosphorus, a nutrient that is essential for the growth of microalgae, is often considered a constraining factor in a variety of aquatic ecosystems and natural environments. The specific composition of the wastewater and the remediation processes employed may cause fluctuations in its presence in wastewater.^51^ In certain instances, the phosphorus levels in wastewater may be sufficient to alleviate its restriction on the proliferation of microalgae.

Microalgae utilize phosphorus for a numerous biological functions, such as the production of ATP (adenosine triphosphate), nucleic acids, phosphate compounds, and phosphorylated enzymes.^52^ Orthophosphate (PO_4_^3−^) and polyphosphates are the primary inorganic compounds used for phosphorus assimilation. The species determines the amount of phosphorus necessary for optimal microalgae growth. Microalgae demonstrate luxury absorption, which involves the accumulation of superfluous phosphorus within their cells when it is readily available. In spite of the scarcity of phosphorus in the environment, microalgae can persist in their development by accumulating phosphorus levels that surpass the minimal requirement.^53^^,^^54^ Microalgae-based wastewater treatment systems have emerged as a symbol of efficiency and sustainability. In microalgae, adsorption is an essential mechanism. Due to their extensive surface area and cell walls that are abundant in lipids, proteins, and polysaccharides, microalgae are capable of adsorbing a diverse array of contaminants that are frequently encountered in wastewater.^55^ Additionally, biosorption is a critical process that entails the passive absorption and accumulation of pollutants in algal detritus, irrespective of whether it is either living or deceased. The intrinsic functional groups, carboxyl, hydroxyl, and amine, significantly improve the biosorption capacity of microalgae cell surfaces, principally in the presence of HMs.^56^

Numerous laboratory experiments have been conducted to illustrate the capacity of various microalgae strains to utilize and eliminate nutrients from wastewater.^57^ For instance, a pilot-scale trial was conducted at a large piggery farm in northern Italy to treat the piggery effluent using an outdoor high-rate algal pond. The average removal efficiencies of nitrogen, orthophosphate, and COD were 90%, 90%, and 59%, respectively, and the biomass productivity was 10.7 ± 6.5 g TSS m^−2^ d^−1^ over 208 days of experimentation.^58^ The primary benefits of this microalgae-based treatment are derived from the fact that the high treatment cost is reduced by the continuous exchange of dissolved and gaseous metabolites between the algal populations and aerobic bacteria, which is achieved by eliminating external aeration.^59^ In conjunction with this energy reduction, it was demonstrated that phycoremediation is effective in reducing both organic and nutrient loads. This process necessitates the use of low-cost solar-powered lagoons or PBRs, which are arranged in a single-step configuration. Another study addresses the primary challenges associated with the use of dairy industry effluents as a nutrient-rich medium in order to achieve high biomass productivity and protein content with *Scenedesmus* sp. that is cultivated in an 80% wastewater-based medium.^60^ Total nitrogen (79.24%) and phosphate (77.14%) exhibited remarkable nutrient removal efficiencies. The proposed culture medium demonstrated its efficacy in fostering substantial biomass and nutritional quality by achieving a maximal productivity of 0.22 ± 0.05 g L^−1^ and a high protein concentration of 384.38 ± 34.06 mg g^−1^.

## Biotechnological applications of biomass

**Figure 2:**
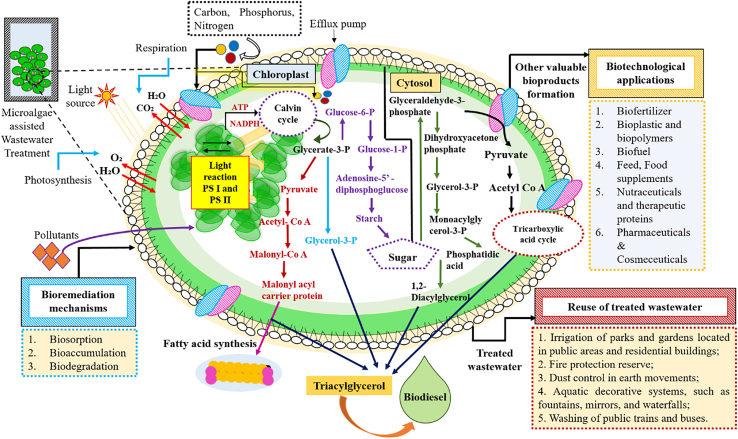
A brief representation of microalgae-assisted wastewater treatment mechanism

An increase in energy consumption, resource depletion, and pollution has resulted from the accelerated expansion of the global population. In order to address these concerns, it is essential to implement environmentally responsible manufacturing and consumption systems that prioritize recycling and reuse opportunities. The biorefinery concept has developed into a sustainable development indicator since its inception. Microalgae cultivation system in wastewater is a promising method that not only improves wastewater remediation but also produces valuable microalgae biomass with a wide range of potential applications, as demonstrated in Figure 2. Microalgae are distinguished by their abundance of bioactive components, which are used in a wide range of commercial applications. Diverse applications encompass their utilization in energy generation, cosmetics, pharmaceuticals, soil biofertilization, and animal feed.

A quick growth and high lipid content of microalgae have prompted interest in their potential as renewable energy sources. In the past decade, microalgae with a high triglyceride content have emerged as a 3^rd^ generation biofuel feedstock.^61^ Triacylglycerol and algal polysaccharides can be converted into bioethanol and biodiesel. The production of biomethane from wastewater-grown microalgae biomass was determined to be influenced by the biomass composition and type of converted molecules.^62^ The sustainability of wastewater could be enhanced by combining the growth of microalgae with anaerobic digestion, which generates CO_2_, and the conversion of methane to energy. A diverse array of conversion techniques, such as pyrolysis, hydrothermal liquefaction, anaerobic digestion and fermentation, and transesterification, are frequently employed to extract energy from microalgae biomass that is cultivated in wastewater. Goswami et al.^63^ Assert that the strain and the kind of energy to be produced are the only factors that determine effective procedures. The high extraction and dehydration costs of microalgae biofuels hinder their commercial viability despite the substantial scientific efforts.^64^ Consequently, microalgae biomass can be employed to manufacture a broader selection of value-added chemicals. A diverse array of beneficial chemicals has been discovered and optimized by marine microalgae. Examples of beneficial molecules with a broad spectrum of biological functions include proteins, fatty acids, polysaccharides, and pigments.^65^

For example, the angiotensin-converting enzyme obtained from the microalgae *C*. *vulgaris* was beneficial in the treatment of hypertension, while the peptide fraction extracted from the microalgae protein detritus digested by pepsin exhibited anti-gastric cancer characteristics. Furthermore, studies have shown that polyunsaturated fatty acids, such as omega-3, have antioxidant qualities, can reduce blood pressure, and can modify the immune system.^66^ Antimutagenic, anticancer, anticoagulant, antimicrobial, anti-inflammatory, and radioprotective activities are exhibited by microalgae pigments, including phycocyanin, lutein, astaxanthin, violaxanthin, and beta carotene.^67^ An additional possible use for microalgae biomass is the conversion of carbohydrates into lactic acid and bioplastics. Glycerol and biodiesel have a volumetric ratio of 10:1 as a result of the transesterification process used in the biodiesel business. For every 1 m^3^ of biodiesel, 0.1 m^3^ of raw glycerol is produced. The growth of the biodiesel sector will lead to a rise in the excess production of glycerol.^68^ Several particular extracts and secondary metabolites from *Dunaliella* sp. and *Tetraselmis* sp. could be used to create a wide range of cosmetics, such as antioxidants, UV-protectants, and anti-aging treatments for the skin.^69^

## Cultivation system for microalgae biomass

### Open and closed cultivation system

The most common form for microalgae production in industrial processes is open ponds, which were originally advocated in antiquity.^70^ They usually consist of long canals in a single- or multiple-loop configuration that are stirred by paddle wheels, or circular ponds with a spinning limb to aid in the mixing of the culture. However, simplified layouts are also feasible. The primary constraints associated with the operation of these open systems are the cost of the harvesting process, the difficulty in sustaining a consistent culture environment, and the inability to regulate contamination. In order to prevent microbial contamination, it is necessary to establish highly selective conditions that guarantee the selected strain dominates the medium. For instance, *D*. *salina* necessitates media that are exceedingly salinous, while *Spirulina platensis* necessitates pH values that are elevated.^71^^,^^72^ Regrettably, neither of these conditions is optimal for the preponderance of microalgae species. Additionally, the direct influence of weather conditions on the properties of open-pond production media renders it exceedingly challenging to preserve the intended environmental parameter values. The harvesting phase results in a significant increase in processing costs as a consequence of the low cell densities that are achieved. This is due to the considerable volume of culture that must be harvested. Consequently, the product’s overall cost experiences a substantial increase. The aforementioned harsh limits have seemingly attained the maximum capacity of open systems, thereby limiting the potential for further technological advancement.

An alternative approach was proposed in response to these challenges, which was predicated on the implementation of closed systems. These are more suitable for sensitive strains that thrive in non-severe environments or when the final product is highly susceptible to degradation by microorganisms (e.g., bacterial metabolization of amino acids and polysaccharides).^73^ The closed construction makes it easier to manage impurities, allowing organisms to flourish in photo-autotrophic, heterotrophic, or mixed modes. Table 1 shows the different microalgae species employed in closed bioreactors for wastewater treatment and product generation. Furthermore, increased cell mass productivities can result in much lower harvesting costs per unit mass (up to three times that of open systems). Nevertheless, the expenses associated with closed systems are significantly higher than those of open systems, and there are numerous other disadvantages.^84^ Despite their enhanced volumetric productivity, closed systems were not a favored industrial choice until recently. This realization is the outcome of high production costs and significant capital expenditure. To lower manufacturing costs, the major elements contributing to the process must be identified and thoroughly examined. This will maximize the benefits and minimize the drawbacks. It is clear that the ideal arrangement is determined by the objective function being assessed. For example, closed systems would be unfeasible for effluent treatment due to the excessive costs involved with huge volumes of feedstock and the minimal added value of the treated material.^85^

**Table 1:** Different microalgae species used in closed bioreactor for wastewater treatment and product formation

S. no	Microalgae	Wastewater	Photobioreactor (PBR)	Treated pollutant	Scale of study	Reference
1	*Chlorella sorokiniana* SU-1	Dairy wastewater	Anaerobic digest PBR	Methane and volatile fatty acids	Pilot scale	Kusmayadi et al.^74^
2	Microalgae natural consortia	Urban wastewater	GreenDune PBR	Ammonia	Pilot scale	Morais et al.^75^
3	*Spirulina platensis* and mixed indigenous microalgae	Urban wastewater	Column PBR	Ammonia	Pilot scale	Almomani et al.^76^
4	*Scenedesmus almeriensis*	Swine wastewater	Thin layer PBR	Tetracycline, ciprofloxacin, and sulfadiazine	Pilot scale	Zambrano et al.^77^
5	*Chlorella vulgaris*, *Scenedesmus obliquus*, *Isochrysis galbana*, *Nannocloropsis salina*, and *Spirulina major*	Sea food effluent	–	Total nitrogen and phosphorus	Full scale	Viegas et al.^78^
6	*Chlorella* sp.	Tannery wastewater	Column PBR	Chromium, cobalt, nickel, cadmium, lead, zinc and copper	Lab scale	Rajalakshmi et al.^79^
7	*Chlorella sorokiniana* and *Scenedesmus acuminatus*	Paddy soaked wastewater	Tubular PBR	Copper and zinc	Pilot scale	Hamed et al.^80^
8	*Chlorella vulgaris*, *Scenedesmus obliquus*, and *Neochloris oleoabundans*	Piggery wastewater	Anaerobic digest PBR	Total carbon, nitrogen, and phosphorus	Pilot scale	Guo et al.^81^
9	*Nannochloropsis oculata*	Oil industry effluent	Tubular PBR	Naphthalene, benzo(*a*)pyrene, benzo(*b*)fluoranthene, and acenaphthylene	Lab scale	Marques et al.^82^
10	Mixed microalgae	Agriculture wastewater	Tubular horizontal photobioreactor	Synthetic musk fragrances tonalide and galaxolide, and the anti-inflammatory drug diclofenac	Full scale	García-Galán et al.^83^

### Types of PBRs

**Figure 3:**
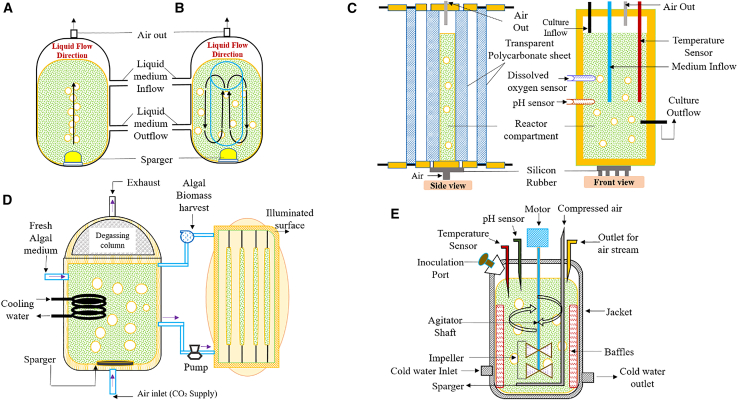
Schematic diagram of conventional PBRs (A) Bubble column. (B) Airlift vertical tubular. (C) Flat panel. (D) Horizontal tubular. (E) Stirred tank.

**Figure 4:**
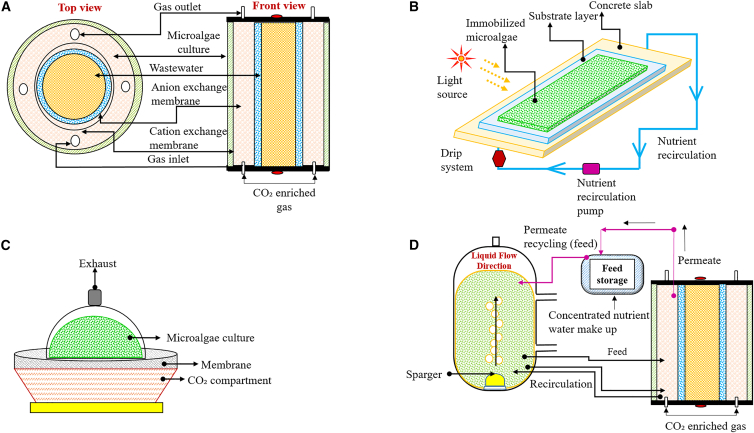
Schematic diagram of non-conventional PBRs (A) Membrane-based. (B) Biofilm PBR. (C) Cell Deg PBR. (D) Hybrid PBR.

Currently, researchers are doing considerable research and development efforts to optimize the design of bioreactors for the generation of algae biofuel.^86^ Microalgae-based biological processes have a considerable influence on PBR design because they play a primary role in biofuel production, as shown in Figures 3 and 4. Light availability has a significant impact on a PBR’s productivity. As a result, a high surface-to-volume ratio is required to guarantee that phytoplankton receive an adequate amount of light and to maximize productivity.^87^

#### Tubular photobioreactors

The most popular kind of closed system made for industrial microalgae cultivation is a tubular PBR. These systems, which are usually made of glass or plastic jars, use pumps or air streams (airlift) to circulate the culture. The Tubular PBRs are broadly categorized into three types: serpentine, manifold, and helical.^88^ The configuration of serpentine and manifold PBRs may be vertical, horizontal, conical, or inclined. The first closed systems to be designed were serpentine reactors. The photostage is a vertically or horizontally oriented planar loop made up of straight tubes joined by U-bends. Two manifolds connect a set of parallel tubes at the extremities of manifold PBRs, one for distribution and the other for culture suspension collection.^89^ Helical PBRs are made up of flexible tubes with small diameters looped around an upright supporting framework. Helical-type systems offer a significant advantage over other PBR categories in that they enable the deployment of tubes that are relatively long over a limited land area. The hydrodynamic stress and cleansing difficulties remain challenging to tackle, as they depend on the microalgae species, flow velocity, and tube diameter.

##### Resource recovery efficiency

Tubular PBRs are among the most extensively researched systems for large-scale applications, as they are suitable for outdoor deployment and have a relatively high surface area to volume ratio.^90^ Nevertheless, their scalability is restricted by the inefficient exchange of gases and the accumulation of oxygen, particularly in long tube lengths. In extended tubular systems, photosynthesis is substantially inhibited by dissolved oxygen buildup. Where CO_2_ mass transfer becomes limiting at high biomass densities, and light penetration is reduced over time by fouling and biofilm formation along tube walls, as highlighted by recent studies.^91^ In addition, the spatial gradients in light exposure and nutrient availability resulting from non-uniform flow dynamics impede scale-up.

##### Practical research gap

There is a lack of design optimization strategies for large-scale tubular systems that simultaneously address oxygen depletion, fouling control, and uniform flow distribution without increasing energy demand.

#### Flat-plate photobioreactors

The cultivation of photosynthetic microalgae has been the subject of extensive research due to the fact that flat plate PBRs have the potential to produce valuable biomass at a low cost in a closed environment with a straightforward design. The simplest configuration is the flat chamber, or more precisely, the simple flat-plate design. Alveolar reactors, however, have also been suggested. The planar panels (sheets) that make up these reactors are separated into a number of internal rectangular tubes (alveoli).^92^ In addition, alveolar reactors have been suggested due to their increased adaptability, more efficient culture flow, higher structural rigidity, and the availability of commercial options at lower construction costs. Standard thicknesses of these sheets are commercially available. In vertical flat-plate and horizontal PBRs, microalgae culture was conducted more frequently.^93^

##### Resource recovery efficiency

The brief light path and high illumination surface of flat-panel PBRs result in superior light utilization efficiency. As a result, they frequently accomplish a higher biomass productivity under controlled conditions than in other configurations.^94^ Nevertheless, their utility at a large scale is restricted by high capital and operational expenses, structural, and material constraints in large installations, warming, and photoinhibition under high irradiance conditions.^95^ Additionally, the maintenance of a consistent light distribution in large panels is a difficult task, particularly in outdoor environments.

##### Practical research gap

Additionally, additional research is required to create scalable panel designs, passive cooling strategies, and cost-effective materials that preserve light-use efficiency without compromising economic feasibility.

#### Airlift and bubble-column photobioreactors

Simple systems called bubble-column and airlift bioreactors are employed in the chemical process, wastewater treatment, and bioprocessing industries. These PBRs with vertical columns are small, inexpensive, and simple to operate. The absence of moving parts, low power depletion, high mass transfer rate, good solids suspension, homogeneous shear, fast mixing, and vertical orientation, which uses less land, are some of the benefits of vertical column PBRs, particularly the airlift PBR, for microalgae cultivation.^96^ However, as the distance from the light source increases, the amount of light that can enter the reactor decreases exponentially, which could be a barrier to the expansion of those bioreactors. Gas bubbles were employed to circulate the culture media in all air-driven PBRs, thereby enabling simultaneous aeration and mingling. Light availability throughout the culture volume can be increased by the right bubble size and spacing, while microalgae metabolic activity and nutritional assimilation are enhanced by gas-liquid mass transfer.^97^ These two primary attributes were identified. In contrast to stirred-tank PBRs, they are capable of accommodating both unicellular and filamentous microalgae due to the reduced shear stress.

##### Resource recovery efficiency

Bubble column and airlift reactors are widely recognized for their efficient mixing, low shear stress, and relatively uncomplicated design, rendering them suitable for sensitive microalgal strains.^98^ In spite of these benefits, they encounter significant obstacles, including restricted light penetration in dense cultures as a result of their larger diameters, diminished photosynthetic efficiency in comparison to flat-panel systems, and the challenge of scaling without compromising light availability.^99^ Despite the fact that hydrodynamics are well-characterized, the interaction between them and light distribution at high cell densities is still not fully understood.

##### Practical research gap

In order to optimize reactor geometry and operational conditions for high-density cultivation, it is necessary to employ integrated light-hydrodynamic modeling approaches.

#### Thin-layer systems

The Institute of Microbiology at Trebon produced microalgae in the 1960s using thin-layer techniques. The use of low-depth/thin-layer cultures to enhance light usage and boost biomass concentration is what sets this system apart.^89^ The retention tank (degasser), where the culture is regulated, and the surface or loop, where photosynthesis occurs, are the two portions of TLSs, which conceptually resemble tubular PBRs. The relative unevenness and incline of the surface determine the culture depth, which is the primary design variable in these systems.

##### Resource recovery efficiency

Due to their high surface-area-to-volume ratio and minimal optical path, thin-layer PBRs (TL-PBRs) demonstrate exceptional light utilization, which leads to high areal productivity.^100^ Nevertheless, their practical application is limited by their susceptibility to contamination, thermal instability, and evaporation losses, particularly in exterior environments. Operational reliability is further compromised by the challenges of maintaining consistent film thickness and uniform flow that are introduced by scale-up.^101^ Additionally, the incorporation of continuous harvesting is still in its infancy.

##### Practical research gap

There is a requirement for closed or semi-closed thin-layer designs that exhibit enhanced thermal regulation, reduced evaporation losses, and controlled hydrodynamics, as well as strategies for seamless integration into continuous bioprocessing systems.

#### Stirred tank system

Previous research has shown that stirred tank PBRs improve microalgae growth, especially for unicellular species, by allowing for effective gas exchange and nutrient distribution.^101^ In order to prevent shear-induced cell injury, filamentous strains may necessitate special precautions. In contrast, microalgae cells typically generate self-shading events, which limit light penetration. The light availability can be increased by improving the light dispersion with internal light sources. Furthermore, they improve photosynthetic activity by adopting efficient CO_2_ transfer through sparging systems.

##### Resource recovery efficiency

Stirred tank PBRs (ST-PBRs) are well-suited for high-value and controlled applications due to their superior control over cultivation parameters and improved mass transfer. However, the extent of their applicability is restricted by the high energy requirements for mixing, the shear sensitivity of microalgae, and the poor light penetration in dense cultures.^102^ In comparison to other PBR designs, their low surface-area-to-volume ratio further restricts efficiency.

##### Practical research gap

Improved internal illumination designs and energy-efficient blending strategies (e.g., intermittent agitation, hybrid airlift-stirred systems) should be the primary focus of future research in order to optimize light distribution while reducing energy utilization.

#### Membrane PBRs

In order to generate microalgae biomass for biofuel production, Roopashri and Makam developed a novel PBR prototype, specifically a hollow-fiber membrane PBR.^103^ By employing a two-stage culture technique, they were able to raise the biomass productivity and lipid concentration in *Tetradesmus obliquus*. Biofilm PBRs have been exploited extensively to increase light discernment for the period of microalgae production where also dropping the land/area imprint of the cultivation system, in addition to the most common PBR designs covered above^104^^,^^105^ offered a unique V-shaped PBR that ensures light is successfully captured and attenuated, addressing the problem of microalgae oversaturation at high light intensities. Numerous additional instances of agricultural methods that are improved through the implementation of innovative technologies exist. A rotating membrane system, a magnet-driven rotary mixing aerator, inclined baffles, a Fibonacci-type vessel, a parallel spiral-flow column, a spiral-ascending CO2 dissolver, an internally illuminated mirror, and other new technological components are all part of these PBRs that have been retrofitted with them.^101^

##### Resource recovery efficiency

Membrane integrated PBRs have garnered attention for their capacity to retain biomass, decouple hydraulic and biomass retention durations, and improve nutrient recovery from wastewater systems.^106^ Nevertheless, their practical application is restricted by the severe membrane degradation that results in increased energy consumption, high operational and maintenance costs, and a lack of long-term stability under continuous operation. Current research suggests that fouling is exacerbated by EPS produced by microalgae; however, there is a lack of effective fouling mitigation strategies.^107^

##### Practical research gap

Self-cleaning systems, low-fouling membrane materials, and hybrid biological integrated physical fouling control mechanisms should be the primary focus of future research.

#### Hybrid and emerging PBRs

Additionally, hybrid PBR systems such as a tubular design merged with an air-lift system, a bubble column connected to a thin lighting platform, or both closed and open raceways have been introduced, combining the advantages of closed PBRs. While air-lift, tubular, and stirred-tank PBRs are more common than others, each has unique features of your own. Because of its effective light exposure, flat-panel PBRs are recommended for small-scale applications and research. They might, however, run into problems and have limited scalability. However, due to their greater mixing capabilities, capacity to adapt to a wide range of scales, and much lower maintenance and operating costs, Airlift PBRs are the best option for commercial high-value commodities.^108^ Furthermore, stirred-tank PBRs are widely used in industry because of their excellent blending capabilities, low maintenance costs, and scalability, which make them the best option for producing biodiesel on a big scale.^109^ Therefore, it is crucial to choose the best design in line with the project’s expected goals and limitations for microalgae production.

##### Resource recovery efficiency

Hybrid PBR systems, such as algae-integrated bacteria consortia reactors, PBR-integrated microbial fuel cell integrations, biofilm, and immobilized-based PBR systems, are being increasingly investigated to improve the efficacy of resource recovery and the robustness of the system.^110^ These systems demonstrate potential for the simultaneous treatment of effluent and the generation of energy, as well as for the improvement of biomass harvesting efficiency and the increased resilience to environmental fluctuations.^111^ However, they continue to be primarily experimental and do not have standardized operational protocols or design.^112^ Furthermore, the inconsistent reporting metrics make it challenging to compare performance across studies.

##### Practical research gap

There is a need of standardized evaluation frameworks and pilot-scale validation of hybrid systems in real-world application.

## Co-culturing strategies for microalgae-assisted treatment

Co-cultivation systems, which combine biology and sustainable industrial practices, can support bioremediation and yield a wide range of products.^113^^,^^114^ The design of these systems demands a thorough understanding of its constituents, whether they are open or contained. The two main types of cultures seen in these systems are axenic cultures (one species), and non-axenic cultures (a large number of microorganisms). The establishment of these co-cultures necessitates the application of evolutionary concepts, including the operation of natural selection on a heterogeneous population (“top-down”) and the selection of genotypes by hand (“bottom-up”).^115^ The circumstances and objectives that have been established determine the potential of both techniques. Biological relationships are as intricate as they are essential. Even when only two species are involved, numerous interactions may occur concurrently in a co-culture system.^116^^,^^117^ For a more comprehensive understanding a co-culture system for microalgae is illustrated in Table 2 for the purpose of effluent treatment and by-product generation. Effective system design necessitates comprehension of these relationships. Co-cultures are available in a variety of forms, such as suspended, flocculated, biofilm, and membrane. The operational methods and design of a reactor are influenced by each of these configurations. The regulation of illumination is an additional critical element. This is particularly critical for co-cultures that are algae-based, as the production of photosynthetic microorganisms is directly influenced by light.

**Table 2:** Microalgae co-cultivation systems for wastewater treatment and byproducts formation

S. no	Co-cultivation system	Culture specifications	Type of wastewater	Application	Product yield	Scale of study	Reference
1	Microalgae and bacteria	*Chlorella vulgaris*, and *Scenedesmus* with filamentous microalgae sp. *Tribonema* and *Lyngbya*	Dairy wastewater treatment	Bioremediation	Lipid	Lab scale	Gupta and Marchetti^118^
2	Microalgae and syntrophic actinomycetes	*Chlorella sorokiniana* and *Streptomyces thermocarboxydus*	Cassava wastewater (biogas effluent)	Bioremediation and bio-product	Biomass and Biodiesel	Lab scale	Padri et al.^119^
3	Microalgae and microalgae	*Chlamydomonas reinhardtii*, *Monoraphidium braunii*, and *Scenedesmus obliquus*	Sewage wastewater	Bioremediation and bio-product	Biomass and Biodiesel	Pilot scale	El-Sheekh et al.^120^
4	Microalgae and microalgae	*Tetraselmis indica* and *Picochlorum* sp.	Municipal wastewater	Bioremediation and bio-products	Lipid, Biodiesel, Astaxanthin, and beta carotene	Pilot scale	Goswami et al.^121^
5	Microalgae and yeast	*Chlorella vulgaris* and *Rhodotorula glutinis*	Starch processing effluent	Bioremediation and bio-product	Lipid	Pilot scale	Lu et al.^122^
6	Fungi-yeast-microalgae	*Penicillium chrysogenum*, *Saccharomyces cerevisiae*, *Chlorella vulgaris*	Livestock wastewater	Bioremediation and bio-product	Bioethanol	Lab scale	Abdalla et al.^123^
7	Fungi and microalgae	The microalga *Tetradesmus obliquus* LCE-01 and the filamentous fungi *Aspergillus niger*, *Penicillium oxalicum*, and *Cunninghamella echinulata*	Petroleum-produced wastewater	Bioremediation	Biomass and Biodiesel	Lab scale	de Andrade et al.^124^
8	Yeast and microalgae	*Rhodotorula mucilaginosa* and *Chlorella vulgaris*	Food-flavorant-producing industry wastewater	Bioremediation	Lipid	Lab scale	Sundaramahalingam and Sivashanmugam^125^

**Figure 5:**
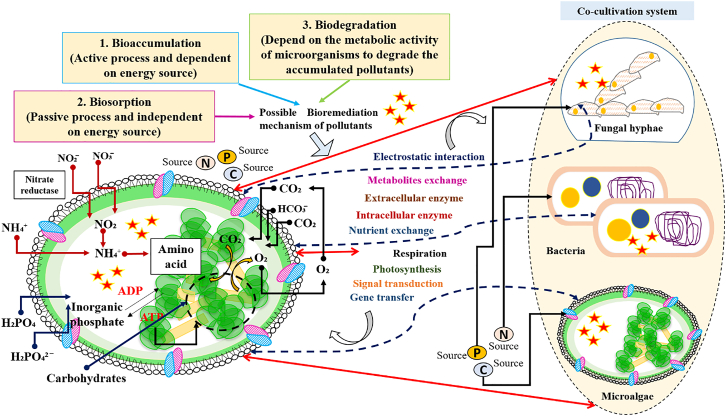
Different bioremediation mechanisms and co-cultivation system associated with microalgae wastewater treatment for enhanced bioproduct formation

Even if little modifications to the co-culturing system may upset its balance, it is crucial to create innovative methods for building vessels, containers, or reactors that can predict culture behavior and aid in future optimization. One important step in the development of consortia production is the identification of the extracellular chemical environment, which comprises the metabolites, peptides, and proteins produced by consortium species.^114^^,^^126^ A brief depiction of different co-cultivation strategies has been showcased in Figure 5.

### Microalgae-bacteria cultivation

The primary characteristics of microalgae and bacteria co-cultures are mutualism and commensalism, with the outcomes being determined by environmental conditions and species compatibility.^44^ Direct mixing, flocculation/pelletization, biofilms, encapsulation, and membrane based systems are among the practicable co-cultivation strategies employed to capitalize on these interactions.^127^ In practice, direct blending has been implemented for biofuel and bioremediation processes resulting in increased metabolite production in comparison to monocultures. Conversely, flocculation based systems improve biomass recovery and harvesting efficiency. Encapsulation, such as alginate beads with cyanobacteria and microalgae systems facilitates biomass reuse and controlled growth environments.^128^ While biofilm cultivation has demonstrated increased light utilization, easier dewatering, and enhanced wastewater treatment efficiency. The experimental dissection of interspecies interactions and the optimization of consortia performance are facilitated by more controlled arrangements, such as membrane separation and dialysis systems, which enable selective metabolite exchange.

In the areas of effluent remediation, bioenergy production, and CO_2_ mitigation, the co-culture systems have been practically implemented. Oxygen and organic carbon are produced by microalgae through photosynthesis, while bacteria provide CO_2_, vitamins (e.g., B_12_), and growth-promoting factors, thereby establishing a self-sustaining cycle.^129^ For example, the co-cultivation of *Chlorella vulgaris* with activated sludge bacteria has improved nutrient removal and dissolved oxygen balance in wastewater systems, while photosynthetic bacteria and green algae have enhanced synthetic wastewater remediation.^130^ In biofuel applications, bacterial partners contribute to the accumulation of lipids for biodiesel, the fermentation of algal carbohydrates into bioethanol, and the enhancement of hydrogen production through metabolite exchange (e.g., acetate and formate transfer).^131^ Furthermore, it has been demonstrated that the methane yields from microalgal biomass can be improved through co-digestion with anaerobic microbes.

Practical challenges persist, such as the stability of consortia under fluctuating conditions, process scalability, and operational costs, despite these promising applications.^132^ Large-scale deployment may be restricted by factors such as oxygen sensitivity in hydrogen production, contamination risks, and variability in metabolite exchange. Consequently, future research should concentrate on the optimization of reactor design, the selection of resilient strains, and the integration of resource recovery systems.^133^ The efficacy and sustainability of microalgae and bacteria consortia can be further enhanced by advancements in biofilm engineering, immobilization techniques, and circular biorefinery approaches, which has established them as a viable solution for next-generation bioenergy and environmental applications.

The co-culture system provides a reliable instrument for the real-time monitoring of algae-bacteria dynamics. The treatment performance and system stability of microalgae-based wastewater processes can be improved by the combination of microfiltration and a high algal inoculum.^134^ A real-time flow cytometry method was devised to simultaneously quantify microalgae *Nannochloropsis oceanica* and bacterial cells (*Paenibacillus sanguinis* and *Bacillus lentus*) that were cultivated on brewery wastewater. The method integrated forward/side scatter analysis, DAPI nucleic acid staining (for bacterial detection), and pigment autofluorescence (as an algal biomarker).^135^ It facilitated the independent monitoring of the dynamics of bacterial and algal populations throughout the cultivation process, as well as the unambiguous discrimination of these populations. Microfiltration effectively removed initial bacterial contaminants while preserving nutrients, allowing a stable algae-bacteria balance.^136^ Conversely, autoclaving resulted in a 31% reduction in ammonium and disrupted advantageous interactions. Bacterial overgrowth resulted in the collapse of the culture in untreated effluent. The microbial balance was maintained and 93%–99% nitrogen and 99% phosphorus removal were achieved by increasing the algal inoculum (OD_750_ from 0.1 to 0.3) in microfiltered medium.

*Chlorella minutissima*, another microalga, has the potential to be a sustainable biorefinery, as it can produce bioactives, lipids, and biomass.^137^ Another study examined the potential of co-cultivation with *Azospirillum brasilense* and *Pseudomonas fluorescens* in low-nitrogen medium to improve productivity, thereby addressing the lack of plant growth-promoting rhizobacteria applications for this alga.^138^ The optimized 1:10 bacterial-algal ratios resulted in 2.5 g L^−1^ biomass (1.6-fold greater than the 1.5 g L^−1^ biomass of the monoculture) and 38% lipid content (1.8-fold greater than the 15%). The bioactives increased considerably, with 45 mg gallic acid equivalents g^−1^ phenolics and 12 mg g^−1^ carotenoids, resulting in 75% radical scavenging and 18 mm antifungal zones against *Candida albicans*. This non-genetic approach surpasses monocultures, providing a scalable, environmentally favorable platform for the remediation of wastewater, biofuels, and nutraceuticals. These findings establish a novel, sustainable biorefinery model, with pilot-scale validation serving as the subsequent phase for industrial application.

### Microalgae-microalgae cultivation

Historically, commercial microalgae cultivation has been characterized by the use of monocultures due to their operational simplicity and ease of control. However, these systems, particularly open ponds, are highly susceptible to contamination by invasive algae, pathogens, and grazers.^139^ The management of such contamination frequently necessitates chemical remedies, which not only increase costs but also present environmental hazards. Conversely, microalgae co-cultivation offers a more sustainable and resilient alternative by simultaneously increasing biomass and lipid productivity, improving nutrient utilization, and enhancing ecological stability.^140^ It is important to note that lipids derived from co-cultures have a higher concentration of short-chain unsaturated fatty acids, which leads to an improvement in biodiesel quality parameters such as viscosity, heating value, and cetane number.

Co-cultivation systems have exhibited robust performance in bioenergy applications and wastewater remediation from a practical standpoint. Bio-flocculation is facilitated by mixed microalgal cultures, which simplifies and reduces operational complexity while facilitating more cost-effective biomass harvesting.^141^ For example, co-culture systems have demonstrated superior removal efficiencies for COD (57%–63%) and total phosphorus (91%–96%) when contrasted with monocultures. In the same vein, research conducted on wastewater PBRs that treat complex effluents (e.g., abattoir wastewater) has reported removal efficiencies of approximately 70% total nitrogen, 96% total phosphorus, and 90% total organic carbon.^142^ In biofuel applications, co-cultivation strategies, such as *Chlorella* with *Monoraphidium*, have substantially improved biomass productivity (up to 62 mg L^−1^ d^−1^), lipid content (∼47%–48%), and overall lipid productivity.^127^ This evidence suggests that these strategies are more economically viable than single-species systems. To achieve the objectives of simultaneous biomass accumulation, biological carbon fixation, and wastewater purification, an investigation into the co-cultivation of *Scenedesmus* sp. 336, *Chlorella sorokiniana* UTEX1602, and *Chlorella* sp. L166 was conducted.^143^ According to the experimental findings, the cell desiccated weight could reach 796.89 mg L^−1^ after setting the mixing ratio of *Scenedesmus* sp. 336 and *Chlorella sorokiniana* UTEX1602 to 1:1 and allowing for 6 h of ventilation per day. Binary microalgae cultures respond metabolically to unfavorable circumstances, such as food constraint, by producing more EPSs due to enhanced cell-cell interactions.

In spite of these benefits, there are specific constraints that must be resolved in order to implement the system on a large scale. Increased EPSs production is frequently the result of improved cell-cell interactions in co-cultures.^144^ While this is advantageous for aggregation, it can impede mass transfer and CO_2_ assimilation if it is excessive. Consequently, it is imperative to implement process optimization and carefully select compatible species. Additionally, it is imperative to develop a more profound mechanistic comprehension of interspecies interactions. The integration of advanced tools, including genome editing, metabolic engineering, and omics approaches, holds significant potential for the decoding of these interactions.^145^ This further enhances the efficiency, stability, and scalability of microalgae co-cultivation systems for sustainable bioenergy production and wastewater treatment.

### Microalgae-fungi cultivation

The co-cultivation of microalgae and fungi has emerged as a cost-effective and promising approach to wastewater remediation and biomass harvesting.^146^ Co-pellets are a significant advantage, as they allow for the efficient and cost-effective recovery of biomass by encasing microalgal cells through electrostatic interactions, protein binding, and exopolysaccharide adhesion in the presence of fungal hyphae.^147^ The consortium functions through complementary metabolic interactions in which microalgae fix CO_2_ through the Calvin-Benson–Bassham cycle, resulting in the production of oxygen and organic compounds. Fungi, on the other hand, utilize these metabolites and release CO_2_ through respiration. This exchange of gases enhances the development of both partners and contributes to substantial reductions in the COD and carbon load of wastewater. Furthermore, the system is significantly more efficient than monocultures due to the enhancement of suspended particulates capture by fungal extracellular enzymes and pellet structures.^148^

Microalgae-fungi systems have exhibited superior nutrient removal efficiencies in practical wastewater treatment applications. *Chlorella vulgaris* and *Ganoderma lucidum* co-cultures exhibit improved phosphorus removal as a result of pH modulation and enzymatic degradation of phosphate compounds.^149^ The presence of functional groups in the cell walls of these consortia also enables the removal of HMs, pharmaceuticals, and pesticides, thereby facilitating adsorption, ion exchange, and complexation. Additionally, the applicability of fungal-assisted systems in complex waste streams is expanded by their ability to withstand high organic loading, a condition in which microalgae alone would typically underperform.

In addition to remediation, the co-cultivation of microalgae and fungi has the potential to generate biofuel and bioproducts. In comparison to monocultures, co-cultures have demonstrated increased biomass accumulation and lipid productivity as a result of metabolic coupling and efficient nutrient exchange.^140^ For instance, microalgae-yeast systems (e.g., *Chlorella* with *Saccharomyces cerevisiae* or *Rhodosporidium toruloides*) have exhibited statistically significant increases in lipid accumulation, biomass yields, and CO_2_ fixation rates.^150^ Fungi have the ability to degrade complex organic substrates using extracellular enzymes and convert them into fatty acids and triacylglycerides. Additionally, they can enhance light penetration through particle formation, thereby promoting algal growth. These systems facilitate the customized production of lipids and fatty acid profiles that are appropriate for biodiesel, as well as other biofuels like bioethanol, biomethane, and biohydrogen.

Large-scale implementation is impeded by a number of obstacles, despite these benefits. The separation of biomass and downstream processing can be challenging due to the strong physical associations and partial hydrolysis of microalgal cell walls.^151^ Strain compatibility and operational parameters, including light intensity, carbon source, and mixing conditions, are also significant factors that influence process performance. Furthermore, although productivity is enhanced by co-cultures, the metabolic interactions that underlie them are not yet entirely comprehended. In order to elucidate these mechanisms, facilitate rational strain selection, and improve scalability, it is imperative that future research incorporate omics approaches (metabolomics and proteomics) and process optimization.^152^ Microalgae-fungi consortia demonstrate substantial potential for the production of next-generation bioenergy, resource recovery, and sustainable wastewater remediation, as they continue to develop.

Microbial contamination is nearly inevitable in wastewater-based cultivation systems due to the naturally occurring presence of a diverse microbial community in wastewater, which includes bacteria, fungi, protozoa, and competing algal species.^153^ Maintaining a stable and controlled interaction is particularly difficult when microalgae are intentionally co-cultured with bacteria, fungi, or other microalgae. The balance between the co-existing organisms is consistently influenced by the environmental conditions of wastewater, including fluctuating nutrient composition, pH, temperature, organic load, and microbial population dynamics.^154^ Consequently, the intended symbiotic relationship can readily transition to overgrowth, predation, or competition with undesirable microorganisms.^155^ Consequently, the system’s stability as a co-cultivation platform is a significant technical challenge and is exceedingly challenging to manage.

In co-cultivation systems between microalgae and bacteria, bacteria frequently facilitate the production of vitamins, the degradation of organic matter, the supply of carbon dioxide, and the recycling of nutrients.^146^ Microalgae, on the other hand, contribute oxygen through photosynthesis. Nevertheless, effluent also introduces opportunistic or pathogenic bacteria that may outcompete beneficial strains for nutrients or produce inhibitory metabolites. Excessive bacterial growth can have a detrimental impact on algal productivity, alter dissolved oxygen levels, and significantly diminish light penetration.^156^ As a result, the optimization of operational parameters, including hydraulic retention time, aeration, nutrient loading, and light intensity, is necessary to preserve the dominance of beneficial bacterial companions and suppress harmful contaminants.^157^

In the same way, fungi enhance biomass harvesting efficacy by facilitating bioflocculation in microalgae-fungi co-cultivation systems. This is achieved through the formation of pellets. However, the growth of microalgae may be diminished as a result of uncontrolled fungal contamination, which may result in parasitic interactions, excessive biomass aggregation, or the consumption of algal-derived organic compounds.^148^^,^^156^ In addition, certain indigenous fungi that are present in effluent may secrete enzymes or secondary metabolites that destroy algal cells.^158^ Consequently, the technical challenge of maintaining a harmonious mutualistic relationship between fungi and microalgae in non-sterile wastewater environments persists.

Multiple algal species are combined in the process of microalgae-microalgae co-cultivation to enhance biomass productivity, environmental adaptability, and nutrient utilization. However, the growth kinetics, nutrient preferences, and light requirements of various algal strains are significantly distinct.^159^ Under wastewater conditions, a single species may swiftly dominate the culture, resulting in the competitive exclusion of other species and a decrease in the overall stability of the system. Furthermore, the control of species and the uniformity of the culture are further complicated by the contamination of effluent by naturally occurring algal species.

Consequently, the technical priorities of large-scale wastewater-based microalgal systems are the prevention of microbial contamination and the preservation of stable interactions among co-cultured organisms.^160^ The current research is increasingly concentrated on the development of robust consortia, adaptive strain selection, environmental control strategies, immobilization techniques, and real-time monitoring approaches to enhance system stability, productivity, and resilience under non-sterile conditions.^132^

## Challenges and future outlook

Despite its potential for use in a wide range of applications, including the production of biofuel, dietary supplements, cosmetics, medicines, and effluent treatment, a number of barriers have made it extremely difficult for microalgae to be commercialized. The most crucial components of microalgae production, PBR setups, are also impacted by these factors.^161^ First, there are concerns regarding the high expense of large-scale microalgae production and cultivation, especially with regard to infrastructure, energy needs for mixing and/or lighting, and nutritional inputs. The economic viability of microalgae-based technologies depends in part on the development of efficient harvesting and processing methods, particularly as most downstream operations are expensive and energy-intensive. High-end PBRs reduce operating expenses and energy consumption by expediting and streamlining the harvesting and dewatering procedures. Due to financial limitations and the requirement for consistent quality and high yields, the transition of microalgae from laboratory to commercial production might be challenging.^86^^,^^162^ Regardless of output amount or PBR configuration, microbial contamination which is mostly brought on by bacteria, protozoa, or fungi causes major performance losses for all microalgae growing systems. These difficulties include the choice and expansion of robust and high-yielding microalgae strains for extensive cultivation, particularly when it comes to the use of genetically modified organisms or food applications, in addition to case-specific regulatory concerns and public perception.^145^^,^^163^ Because of its rapid progress and remarkable capacity to tackle real-world problems, AI has attracted a lot of interest in recent years and is presently being actively applied in a number of industries.^164^ In recent years, there has been an increasing interest in the application of AI-based models to predict complex interactions in microalgae cultivation systems. Artificial neural networks (ANNs) and convolutional neural networks have demonstrated effectiveness in tasks such as biomass quantification, genome interaction prediction, and flocculation behavior, thereby reducing the experimental burden and enabling process optimization.^165^ These models present a promising alternative for capturing the complex interplay between the characteristics of wastewater, the proliferation of microalgae, and the accumulation of intracellular metabolites, particularly carbohydrates. In parallel, traditional computational and kinetic models, which are primarily based on Droop and Monod formulations, have been extensively employed to forecast biomass growth and lipid production under single-variable conditions (e.g., carbon, nitrogen, phosphorus, or light).^166^ Meanwhile, more sophisticated multi-parameter models have integrated factors such as total inorganic carbon, nutrients, light, and temperature to simulate microalgal dynamics in both open and closed systems, including mixotrophic growth scenarios with simultaneous carbohydrate and lipid accumulation. ANNs are computational models that are derived from the structure and functionality of biological neural networks.^167^ They are instructed on historical data to develop an understanding of the intricate relationships between input and output variables. ANNs have been employed to predict and optimize a variety of microalgae cultivation parameters, such as biomass production, nutrient uptake, and effluent treatment efficiency. The prediction of biological nutrient removal in municipal wastewater using *Neochloris oleoabundans* within batch PBRs was recently achieved through the application of tree-based machine-learning techniques, including decision tree regressor and extra tree regressor.^168^ As a result of their capacity to manage high-dimensional datasets and complex, nonlinear relationships, tree-based models, including decision trees, random forests, and gradient-boosting machines, have become increasingly popular in environmental modeling. Ensemble models are a potent machine learning method that enhances the overall accuracy and robustness by combining the predictions of multiple individual models. Ensemble methods can mitigate the risk of overfitting and improve predictive performance by combining the outputs of multiple models.^169^ Numerous applications, such as biotechnology, environmental sustainability, and waste management, could be significantly advanced by photo-bioreactors for microalgae culture. Several important difficulties need to be addressed and considered in the next iteration of industrial-scale PBR designs. The implementation of AI/ML in microalgae systems raises severe ethical and operational concerns regarding data governance, bias, accountability, and transparency.^170^ Trust in decision-making is compromised, particularly in environmentally sensitive and safety-critical processes, due to the limited interpretability of black-box models. Dataset bias, which is frequently the result of geographically or biologically constrained training data can impede model generalization and result in suboptimal or inequitable outcomes.^171^ While liability for erroneous predictions remains ambiguous, integration with IoT introduces additional challenges in data privacy, ownership, and cybersecurity. Data scarcity and noise, high computational costs, lack of standardization, and challenges in scaling models for industrial applications are among the primary technical obstacles. Continuous model adaptation is required due to biological variability, fragmented data infrastructures, and dynamic process conditions.^172^ Furthermore, it is imperative to conduct a thorough evaluation of the environmental trade-offs associated with energy-intensive AI computations in order to guarantee that there are positive sustainability benefits. Stronger regulatory oversight and interdisciplinary capacity building are necessary to address these issues, in addition to explainable AI frameworks, robust validation protocols, adaptive learning systems, and standardized methodologies.

## Conclusion

The development, production, and utilization of biofuels derived from microalgae are instrumental in the achievement of substantial SDGs from the 2030 Agenda. This analysis illustrates that the production of microalgae biofuels on a large scale is confronted with numerous obstacles, particularly in the realm of economics. The efficacy and performance of PBRs can be enhanced by incorporating them with other emergent technologies, such as AI and machine learning. These digital systems have the capacity to analyze complex data, predict the most optimal production conditions, and automate real-time adjustments. It is inevitable that technological advancements will continue to influence the landscape of sustainable bioprocessing and resource utilization, resulting in the introduction of intriguing industrial-scale PBR potential. In order to guarantee economic viability and process efficiency, future research should concentrate on optimizing economical feedstock selection, improving harvesting techniques, and systematically evaluating key operational parameters, including pH, temperature, light intensity, nutrient availability, and carbon sources. It is crucial to note that the efficacy of these systems is contingent upon the interactions between microalgae and their associated microbial partners. These synergistic relationships substantially improve productivity and functional stability. In this context, the development of omics technologies provides potent tools to decode intricate interspecies interactions and metabolic networks within co-cultures, thereby generating insights that can be used to drive further optimization. In conclusion, the strategic application of emergent analytical technologies, precise control of operating conditions, and an integrated system-level understanding are essential for the sustainable industrial-scale production of valuable bioproducts from microalgal co-cultivation systems.

## Acknowledgments

The authors declare that no funds, grants, or other support were received during the preparation of this manuscript.

## Author contributions

Conceptualization and writing – original draft, A.S., V.M., and D.R.S.; visualization, A.S.; writing – review and editing, V.M. and D.R.S.

## Declaration of interests

The authors declare no competing interests.
